# Upas Tree

**Published:** 1846-12

**Authors:** 


*Upas Tree.*—The Dyak Darrat or Land Dyaks, seems to differ in no essetial particular of language or costumes, from the men of the sea, except in as far as depends on their inland position. The only remarkable difference of usage noticed by Mr. Brooke is, that the latter use and the former do not, the weapon called the *sumpitan*, or blowpipe, for shooting poisoned arrows. The wounds inflicted by these are curable, says Mr. Brooke, by antidotes known to the natives; nor are they regarded, apparently, with much terror. And we suspect the whole romantic history of the poisonous trees of the Indian isles must be banished with so many other marvels, to the province of legends, since a friend of Mr. Davidson, in Java, to prove their absurdity, climbed up an upas tree and passed two hours in its branches, where he took his lunch and smoked a cigar.—*Edinburgh Rev.*—*Ibid.*

